# The impact of digital communication and offline social interactions on depressive symptoms in Chinese older people

**DOI:** 10.3389/fpubh.2024.1387637

**Published:** 2024-11-13

**Authors:** Qi Chai, Zhengting Yang, Yiting Luo, Yin Deng, Lu Qin, Zhibo Yang, Ruizhi Wang, Yongzhao Zhou

**Affiliations:** ^1^Integrated Care Management Center, Institute of Respiratory Health, West China Hospital, Sichuan University, Chengdu, China; ^2^Frontiers Science Center for Disease-related Molecular Network, West China Hospital of Sichuan University, Chengdu, China; ^3^Department of Respiratory and Critical Care Medicine, West China Hospital of Sichuan University, Chengdu, China

**Keywords:** digital communication, offline social interaction, depression symptoms, older people, pandemic, propensity score matching

## Abstract

**Background:**

In a special period of lack of offline social interaction (pandemic), the mentality of older people is changing quietly. This study aims to dissect the impact of these changes on their mental health.

**Method:**

Utilizing data from the China Health and Retirement Longitudinal Study (CHARLS 2020), this research included 7,784 participants aged over 60 years. It evaluated the prevalence of depressive symptoms, and assessed the relative effects of these interactions on depressive symptoms.

**Results:**

(1) a depressive symptom prevalence of 40.65%; (2) a modest engagement in digital communication, with only 20.39% of the older participating; (3) varying prevalences of depressive symptoms across groups, with notable differences depending on the type and combination of social interactions. Specifically, the prevalence was 21.7% among those engaging only in digital communication, and varied from 21.7 to 32.0% among other groups, highlighting the significant impact of social interaction patterns on depressive symptoms. Statistical analysis confirmed the significance of these findings (*χ*^2^ = 42.415, *p* < 0.001). (4) In the first model, digital communication was associated with a lower likelihood of depressive symptoms (OR = 0.820, 95%CI: 0.707–0.950, *p* < 0.05). The second model showed no significant effect of offline social interactions on depressive symptoms (OR = 0.124, 95%CI: 0.917–1.143, *p* = 0.678). The third model demonstrated that the inclusion of offline social interaction variables did not significantly alter the beneficial effect of digital communication (OR = 0.820, 95%CI: 0.707–0.952, *p* < 0.05). (5) The results of the 1:1 matching analysis indicated that digital communication significantly alleviated depressive symptoms in older people (ATT = −0.054, *p* < 0.05). Conversely, offline social interactions did not significantly affect the depressive symptoms of this demographic (ATT = −0.028, *p* > 0.05).

**Conclusion:**

The depressive symptoms among older people in China has greatly increased during 2020 which year the COVID-19 pandemic was in the early stage, accompanied by considerable changes in their modes of social interaction. Our findings suggest that the influences of digital communication and offline social interactions on depressive symptoms may have operated independently during the pandemic. The potential of digital communication engagements in enhancing mental health, particularly in exceptional circumstances such as during a pandemic, underscores the need for further exploration.

## 1 Introduction

During the 13th Five-Year Plan period (2016–2020), China established the world’s most extensive optical-fiber and 4G network, achieving connectivity in over 99 percent of its villages. This infrastructure supported the proliferation of 4G base stations, which comprised over half the global total. A noteworthy trend over the last decade has been the marked increase in older users within social media, with the number of internet users aged 60 and above reaching 119 million by 2021 (1). As China grapples with demographic shifts and an aging population, the number of older internet users is expected to sharply increase in the coming years.

Digital communication is characterized by social media platforms that enable users to connect and engage with others through various means (2). Such platforms facilitate two-way connections and interactions with friends, support audio and video communications, and allow users to share and consume content provided by their connections (3). Given the popularity of WeChat in China, our research primarily focuses on this platform. Additionally, the survey year for our data was 2020, coinciding with the second year of the COVID-19 epidemic in China.

To mitigate the spread of the virus, the Chinese government implemented comprehensive lockdown policies in various cities starting in January 2020. The pandemic, coupled with strict social distancing measures, generated unprecedented stressors, including perceived severe threats to personal safety, intense fears, and strong feelings of being out of control (4). With offline social interactions restricted during the quarantine period, digital communication became the primary channel for people to acquire relevant information, basic supplies, and a sense of belonging. However, the relationship between the use of digital communication platforms and mental health remains controversial. While studies have indicated that social media use can decrease life satisfaction and increase self-harm, suicidal ideation (5–7), psychological distress, depression, and anxiety (5), most of these studies originate from Western countries, with only a few conducted in Asian contexts (5–7). Conversely, certain studies highlight the benefits of digital communication, such as promoting health behavior changes, obtaining information support, and maintaining connections with others (8, 9), which were crucial during the pandemic.

Prior studies indicate that the impact of digital communication on mental health is complex, varying significantly based on factors such as patterns of social media use (6). Much of the existing research on social media use and psychological well-being has focused on healthy students and adolescents, overlooking other demographic groups such as older adults. It is important to note that the media socialization patterns of older and younger people are distinctly different. According to the Socioemotional Selectivity Theory, older adults prioritize emotional fulfillment in their relationships and typically favor close social ties over weaker ones (10). In contrast, younger individuals often use social interactions to develop new ties and expand their social networks (10). Consequently, it may be inappropriate to apply existing research findings on digital media use and the mental health of young people directly to older adults.

During the COVID-19 pandemic, a cross-sectional survey revealed that risk perception was heightened among older adults, who were more likely to view indoor activities as unsafe (11). For older people, the decision to engage in social contact through certain modes may depend on the availability of social ties and their comfort with various communication methods. The social compensation hypothesis posits that online communication can serve as an alternative mode of contact when in-person interactions are unavailable (12). Based on this understanding, we propose two hypotheses: (1) Offline social interactions may be perceived as unsafe during the pandemic, diminishing their protective effect on the mental health of older adults; (2) Digital communication may offer a safer alternative for older people to socialize, potentially compensating for the lack of in-person interaction and thus exerting a protective influence on their mental health.

Exploring the impact of digital communication on the mental health of older adults not only advances research in this field but also enhances our understanding of how digital communication can complement traditional social interactions during this critical period of the pandemic. Given the increasing age of the global population, this research may hold significant potential for further study.

## 2 Methods

### 2.1 Data sources

This research draws on data from the China Health and Retirement Longitudinal Study (CHARLS 2020), a comprehensive, longitudinal survey targeting residents aged 45 and above across China, spanning from 2008 to 2020. CHARLS aims to collect a wide range of data, including demographic characteristics, health status and functionality, socio-economic status, and retirement-related information. Employing a multistage probability sampling technique, the CHARLS team has successfully conducted surveys among middle-aged and older individuals in 28 provinces, resulting in five waves of national surveys in 2011, 2013, 2015, 2018, and 2020 (13, 14). The present study utilizes the 2020 survey data, which initially included 19,335 participants. Focusing on respondents aged 60 and above reduced this number to 10,623. After excluding cases with missing values for key variables, the final sample comprised 7,784 respondents. It is noteworthy that the fifth survey commenced in early 2020. Due to the outbreak of COVID-19 at the end of 2019, intermittent home isolation and lockdown policies were implemented across most parts of China throughout 2020. During this survey, investigators overcame various challenges, conducted intermittent surveys in compliance with the epidemic prevention and control policies of different regions, and successfully completed the fifth wave of data collection by the end of 2020.

### 2.2 Measurement

This study mainly primarily examines the association between digital communication and offline social interactions and their impact on depression among older people. Recognizing the complexity of this relationship, the study also considers a range of factors previously identified as influential. Research has highlighted that gender, Hukou (household registration) status, age, education level, marital status, quality of family relationships, health-related behaviors, objective health status, and economic income can affect both social interactions and depression (15–19). These variables have been known to mediate or modify the effects of social interaction on depression. Accordingly, to isolate the specific impact of digital communication and offline social interactions on depressive symptoms, we have controlled for these factors in our analyses.

#### 2.2.1 Dependent variables

Depressive symptoms were evaluated using the 10-item Center for Epidemiologic Studies Depression Scale (CESD-10) in the latest wave of the study. This scale, a streamlined version of the original 20-item CESD, omits items considered highly redundant to improve efficiency without compromising diagnostic accuracy. Respondents rated the frequency of specific feelings over the past week on a scale from 0 to 30, with scores from 0 to 9 suggesting an absence of depressive symptoms and scores of 10 or higher indicating the presence of depressive symptoms. The CESD-10 is highly validated for use in general populations and has demonstrated both reliability and validity specifically within community-dwelling older populations in China (20).

#### 2.2.2 Independent variables

Digital communication: We identified participants who had engaged in internet use within the past month. To differentiate general online behavior from online communication behavior, we classified individuals as engaging in digital communication if they participated in at least one of the following activities: online chatting, posting on social platforms (e.g., WeChat), or using social media apps such as WeChat for communication.

Offline social interaction: This was assessed based on the participants’ involvement in at least one of the following activities in the past month: visiting or socializing with friends, playing mahjong, chess, cards, or participating in community activity room events, offering assistance to relatives, friends, or neighbors not residing with them, engaging in physical activities such as dancing, exercising, practicing qigong, involvement in community organization activities, volunteering or charitable endeavors, caregiving for a sick or disabled individual not living with them, attending school or training courses, and other unspecified social activities.

Based on participation in these activities, the respondents’ offline social interactions were categorized into three distinct groups for analytical clarity: those with no offline social interactions, those engaged in one type of offline social interaction, and those involved in two or more types of offline social interactions.

#### 2.2.3 Covariates

1. Relationship satisfaction with children: To gauge satisfaction levels in parent–child relationships, participants were asked, “Are you satisfied with your relationship with your children?” Responses ranged from “not satisfied” to “extremely satisfied,” and scored on a scale from 1 to 5 points. A higher score indicated greater satisfaction with the relationship.
2. Daily life self-care ability: The self-care abilities of the older people were assessed using a combined approach of the Activities of Daily Living (ADL) scale and the Instrumental Activities of Daily Living (IADL) scale, encompassing 11 items in total. These items were categorized into three responses: “I have difficulty and need help,” “I have difficulty but can complete,” and “no difficulty,” scored from 1 to 3 points, respectively. The aggregate score ranged from 11 to 33 points, with a higher score reflecting better self-care ability.
3. Income: Household income was calculated by summing the personal income of respondents and their spouses, other family members’ incomes, and transfer or supplementary income (e.g., pensions, government aid). To mitigate the impact of extreme values and reduce data variability, household income was logarithmically transformed for the analysis. The details of all variables are provided in Table 1.

**Table 1:** The study variables with their respective definitions.

Variable	Definitions
Depressive symptoms	0 = have on depression symptom;1 = have depression symptom
Digital communication	0 = have no digital communication;1 = have digital communication
Offline social interaction	0 = have no offline social interaction;1 = have only one social interaction;2 = have two or more social interaction
Type of offline social interaction	The total score is 0–8, with higher scores indicating more participation in offline social interaction categories
Gender	0 = male;1 = female
Hukou	0 = rural;1 = urban
Age	Quantitative variables, ≥60 years old
Education	0 = primary below;1 = primary or junior;2 = senior;3 = senior above
Marriage	0 = Separated, divorced, widowed, unmarried;1 = married
Relationship satisfaction with children	The total score is 1–5, with higher scores indicating higher satisfaction
Smoking	0 = no smoking;1 = smoking
Drinking	0 = no drinking;1 = drinking
Chronic diseases	0 = no chronic disease;1 = have chronic disease
Daily life self-care ability	The total score is 11–33, with higher scores indicating better self-care
Household income	Quantitative variables, take log values

### 2.3 Statistical analysis

Statistical analysis was conducted using Stata 15. For descriptive statistics, continuous variables following a normal distribution were presented as means ± standard deviations (X ± S), and the independent samples t-test was used to compare between two groups. Categorical variables were expressed in terms of frequencies and percentages, with the chi-square test utilized for group comparisons.

The analysis commenced with a chi-square test to identify overall differences in depressive symptoms across five social interaction categories among older people. The investigation then proceeded to logistic regression analysis to explore the effects of digital communication and offline social interactions on depressive symptoms in older people. Three models were employed: the first model assessed the impact of digital communication without considering offline social variables; the second model focused on offline social interactions by excluding digital communication variables, to gauge the influence of offline interactions on mental health; and the third model incorporated both digital communication and offline social interaction variables to evaluate whether these types of social interactions independently or jointly influence depressive symptoms among the older people.

To ensure the robustness of our findings, we applied propensity score matching (PSM) subsequent to multivariate analysis. This involved using 1:1 matching, 1:4 matching, and kernel matching to assess the reliability of the health effects associated with various forms of social interaction.

## 3 Result

### 3.1 Demographic characteristics

In the survey of 7,784 older individuals, 3,205 (40.65%) exhibited depressive symptoms. 1,587(20.39%) having digital communication. Our analysis revealed significant differences between older individuals with depressive symptoms and those without, across several variables, including the extent of digital communication and offline social interactions, gender, Hukou status (registered permanent residence), marital status, education level, smoking habits, alcohol consumption, satisfaction with children’s relationships, presence of chronic diseases, and daily life self-care abilities (*p* < 0.05; Table 2).

**Table 2:** The basic information of the respondents.

Variable	Total (%/X ± S)	Having depression symptom (*n* = 3,205)	No depression symptom (*n* = 4,579)	t/*χ*^2^	*p*
Digital communication				78.949	<0.001
Yes	1,587 (20.39)	498 (31.4)	1,089 (68.6)		
No	6,197 (79.61)	2,707 (43.7)	3,490 (56.3)		
Offline social interaction	0.66 ± 0.90	0.62 ± 0.837	0.69 ± 0.941	3.367	0.001
Age	68.25 ± 6.20	68.35 ± 5.997	68.19 ± 6.339	−1.148	0.251
Gender				208.835	<0.001
Male	4,023 (51.68)	1970 (49.0)	2,053 (51.0)		
Female	3,761 (48.31)	1,235 (32.8)	2,526 (67.2)		
Hukou				94.839	<0.001
Urban	6,480 (83.24)	2,826 (43.6)	3,654 (56.4)		
Rural	1,304 (16.74)	379 (29.1)	925 (70.9)		
Marriage				53.060	<0.001
Married	6,140 (78.88)	2,399 (39.1)	3,741 (60.9)		
Separated, divorced, widowed, unmarried	1,644 (21.12)	806 (49.0)	838 (51.0)		
Education				157.244	<0.001
Primary below	4,281 (54.99)	1998 (46.7)	2,283 (53.3)		
Primary or junior	2,833 (36.39)	1,047 (37.0)	1,786 (63.0)		
Senior	576 (7.39)	140 (24.3)	436 (75.7)		
Senior above	94 (1.21)	20 (21.3)	74 (78.7)		
Smoking				98.102	<0.001
Yes	3,507 (45.05)	1,230 (35.1)	2,227 (64.9)		
No	4,277 (54.94)	1,975 (46.2)	2,302 (53.8)		
Drinking				81.848	<0.001
Yes	2,546 (38.39)	2,341 (44.7)	2,897 (55.3)		
No	5,238 (61.61)	864 (33.9)	1,682 (66.1)		
Relationship satisfaction with children	3.53 ± 0.74	3.63 ± 0.682	3.38 ± 0.794	14.718	<0.001
Chronic disease				132.053	<0.001
Yes	6,722 (86.36)	2,939 (43.7)	3,783 (56.3)		
No	1,062 (13.64)	266 (25.0)	796 (75.0)		
Daily life self-care ability	30.67 ± 2.84	31.51 ± 3.00	29.91 ± 1.69	−10.77	<0.001
Household income (logarithm)	9.48 ± 1.38	9.18 ± 1.34	9.68.36 ± 1.38	15.278	<0.001

### 3.2 Prevalence of depressive symptoms across social interaction groups

Participants were categorized into five groups based on their social interaction patterns: Group 1 consisted of individuals engaging exclusively in digital communication; Group 2 included those with both digital communication and offline social interactions; Group 3 comprised individuals without any social interactions and digital communication; Group 4 involved participants with only one form of offline social interaction; and Group 5 consisted of those with two or more types of offline social interactions.

Our findings revealed varying prevalence rates of depressive symptoms across these groups: 26.2% in Group 1 (159 out of 607), 21.7% in Group 2 (213 out of 980), 32.0% in Group 3 (1,173 out of 3,670), 29.5% in Group 4 (522 out of 1770), and 29.1% in Group 5 (220 out of 757). A significant overall difference in depressive symptom prevalence was observed among the five groups (*χ*^2^ = 42.415, *p* < 0.001; Table 3).

**Table 3:** Differences in depressive symptoms among older adults with different social behaviors.

Group	Total (%)	Having depression symptom	No depression symptom	*χ* ^2^	*p*
Group 1	607 (7.79)	159 (26.2)	448 (73.8)	42.415	<0.001
Group 2	980 (12.59)	213 (21.7)	767 (78.3)		
Group 3	3,670 (47.15)	1,173 (32.0)	2,497 (68.0)		
Group 4	1,770 (22.74)	522 (29.5)	1,248 (70.5)		
Group 5	757 (9.73)	220 (29.1)	537 (70.9)		
Total	7,784	2,287	5,497		

### 3.3 The influence of older people depression symptom binary logistics regression analysis

In Model 1, where offline social interaction variables were omitted, participants engaging in digital communication demonstrated a reduced likelihood of experiencing depressive symptoms (OR = 0.820, 95% CI: 0.707–0.950, *p* < 0.05). Furthermore, being male, having an urban household registration, being married, reporting higher satisfaction with child relationships, possessing better activities of daily living, and having a higher family income were associated with a lower risk of depressive symptoms (all OR < 1, all *p* < 0.05). Older individuals with chronic diseases were more likely to exhibit depressive symptoms compared to those without chronic conditions (OR = 1.978, 95% CI: 1.693–2.311, p < 0.05).

In Model 2, exploring the impact of offline social interactions on psychological health, no statistically significant association was found between offline social interaction and depressive symptoms among older people (OR = 1.024, 95%CI:0.917–1.143, *p* > 0.055).

In Model 3, where both digital communication and offline social interaction variables were included, the coefficient for digital communication remained unchanged (OR = 0.820, 95%CI: 0.707–0.952, *p* < 0.05). While the inclusion of offline social interaction somewhat altered the influence coefficients of other variables on depressive symptoms, the overall positive and negative effects remained consistent (Table 4).

**Table 4:** Binary logistic regression analysis of the influence of digital communication and offline social interaction.

		Model 1		Model 2		Model 3	
Variables	Control group	OR (95%CI)	*p*	OR (95%CI)	*p*	OR (95%CI)	*p*
Having digital communication	No digital communication	0.820 (0.707,0.950)	0.008			0.820 (0.707,0.952)	0.009
One type of offline social interaction	No offline social interaction			1.024 (0.917,1.143)	0.678	1.071 (0.969,1.185)	0.180
Two or more types of offline social interaction				1.094 (0.950,1.259)	0.214	0.652 (0.391,1.087)	0.101
Male	Female	0.587 (0.503,0.686)	<0.001	0.591 (0.507,0.689)	<0.001	0.592 (0.506,0.692)	<0.001
Urban	Rural	0.808 (0.689,0.948)	0.009	0.719 (0.614,0.841)	<0.001	0.805 (0.686,0.944)	0.008
Married	Separated, divorced, widowed, unmarried	0.862 (0.758,0.981)	0.024	1.138 (1.010,1.283)	0.034	0.028 (0.865,0.760)	0.008
Primary or junior	Primary below	0.954 (0.851,1.069)	0.954	0.934 (0.836,1.044)	0.231	0.953 (0.850,1.068)	0.405
Senior		0.690 (0.548,0.870)	0.690	0.695 (0.555,0.869)	0.001	0.701 (0.556,0.884)	0.701
Senior above		0.760 (0.440,1.312)	0.760	0.713 (0.412,1,235)	0.227	0.782 (0.452,1.352)	0.378
Smoking	No smoking	1.029 (0.890,1.191)	0.698	1.012 (0.877,1.169)	0.866	1.025 (0.886,1.186)	0.743
Drinking	No drinking	0.906 (0.808,1.017)	0.094	0.914 (0.815,1.024)	0.121	0.903 (0.804,1.013)	0.083
Relationship satisfaction with children		0.619 (0.578,0.663)	<0.001	0.620 (0.580,0.664)	<0.001	0.619 (0.577,0.663)	<0.001
Having chronic disease	No chronic disease	1.982 (1.697,2.316)	<0.001	1.977 (1.695,2.306)	<0.001	1.978 (1.693,2.311)	<0.001
Activity of daily life		0.877 (0.860,0.894)	<0.001	0.879 (0.863,0.896)	<0.001	0.876 (0.859,0.893)	<0.001
Household income (logarithm)		0.840 (0.806,0.875)	<0.001	0.841 (0.811,0.877)	<0.001	0.839 (0.806,0.874)	<0.001
Constant		2998.305	<0.001	2994.118	<0.001	2986.019	<0.001

### 3.4 Robustness analysis

**Figure 1:**
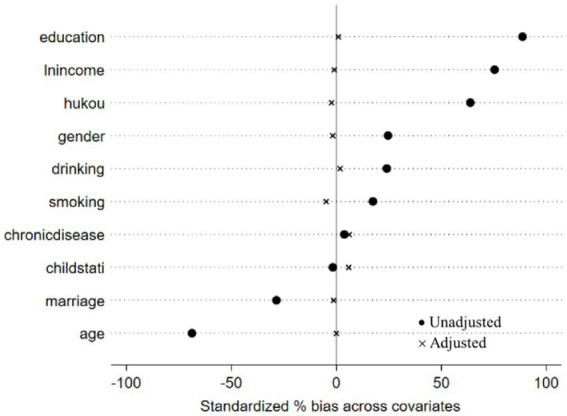
Two groups of digital communication or not.

**Figure 2:**
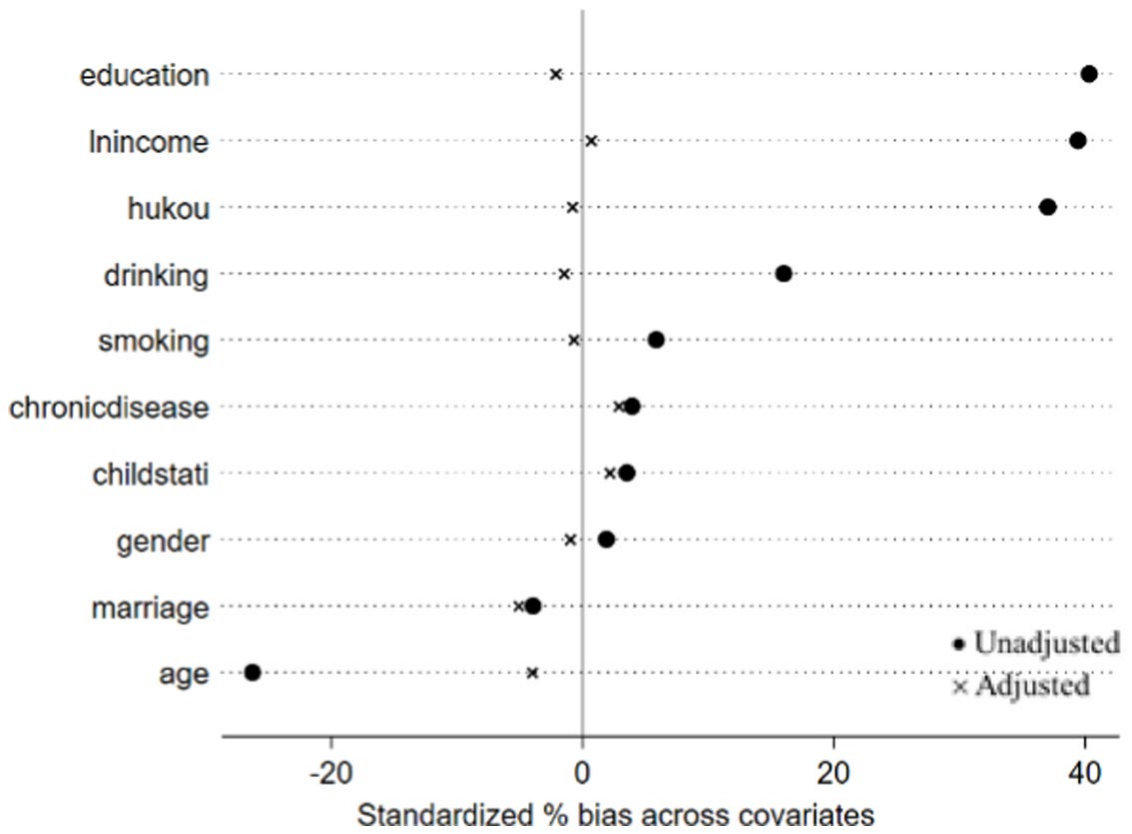
Two groups of offline social interaction or not.

In the analysis presented in Table 4, no statistically significant association was found between offline social interaction and depressive symptoms in older people. This finding notably contrasts with results from previous studies. For the multivariate analysis, we included only statistically significant indicators as matching variables. PSM was utilized to balance the distribution of these variables between the two groups. To ascertain the robustness of this conclusion, we further validated the results using PSM. The efficacy of the matching process was evaluated by examining the absolute standardized differences (ASD) before and after PSM, as illustrated in Figures 1, 2.

In propensity score matching, the average treatment effect on the treated (ATT) quantifies the difference in depressive symptoms between older adults engaged in digital communication or offline social interaction and those who are not. Utilizing a 1:1 matching approach, the results indicated that: (1) the influence of digital communication on depressive symptoms was statistically significant (*ATT* = −0.054, *p* < 0.05); (2) offline social interaction had no statistically significant effect on depressive symptoms (*ATT* = −0.028, *p* > 0.05), as shown in Table 5.

**Table 5:** The exploration effect of social interaction on depressive symptoms in older people based on PSM.

Matching methods	Digital communication		Offline social interaction	
	ATT	*t*	ATT	*t*
1:1 matching	−0.054	−2.14	−0.028	−1.65
1:4 matching	−0.047	−2.27	−0.019	−1.35
Kernel Matching	−0.051	−2.75	−0.026	−2.01

To mitigate the bias introduced by the matching method, we employed 1:4 matching and kernel matching to verify the results. The results showed that digital communication continued to alleviate depressive symptoms in older people across different matching methods (1:4 matching: *ATT* = −0.047, *p* < 0.05; kernel matching = −0.051, *p* < 0.05). Conversely, offline social interaction still exhibited no significant impact on depressive symptoms (1:4 matching: *ATT* = −0.019, *p >* 0.05; kernel matching = −0.026, *p* > 0.05), as shown in Table 5.

## 4 Discussion

Our study revealed a high prevalence of depressive symptoms among older people, with 40.65% exhibiting such symptoms, which is a stark contrast to the findings from the CHARLS survey before (21, 22). We attribute this notable increase to the pervasive impact of the COVID-19 pandemic in China. Our research data were collected in 2020, during the early stages of the epidemic when no vaccine had been developed and public knowledge about the novel coronavirus was still limited. This period likely led to significant psychological distress among China’s older population, contributing to the observed rise in depressive symptoms; a trend consistent with numerous studies highlighting the pandemic’s adverse effects on mental health (23, 24).

Our investigation delved into the impact of “digital communication” on the mental well-being of older individuals, exploring the effects of emerging socialization methods on depressive symptoms. Despite our findings indicating a relatively modest proportion of older individuals (20.39%) engaging in digital communication, this figure marks a noteworthy improvement over the CHARLS2018 data (11.3%) (25). However, it is important to acknowledge the potential underestimation of digital communication prevalence among older people. Reports indicate that by December 2021, approximately 90% of Chinese citizens were registered users of digital communication apps such as WeChat (1). In early 2020, China’s epidemic prevention and control policies mandated the use of an electronic health code for all individuals in public areas. WeChat, serving as a key platform for the “health code,” likely saw a surge in registrations. However, this increase in registrations does not necessarily imply that all registered users actively utilized WeChat as a primary tool for frequent communication. It is possible that many registered simply to display their health code (26). Going forward, we believe the most appropriate approach is to continuously track future CHARLS survey data. By comparing the changes in the proportion of the number of people who had digital communication each time, we may be able to judge the rationality of 20.67% of the data in this paper.

Our single-factor analysis revealed that older individuals who engaged in digital communication exhibited a lower proportion of depressive symptoms compared to those without digital communication. Furthermore, those participating in both digital communication and offline social interaction displayed a lower prevalence of depressive symptoms compared to those solely engaged in digital communication. These findings suggest a potential protective role of offline social interaction in older people’s mental health. However, when incorporating offline social interaction into our multifactor model assessing the impact of digital communication on depressive symptoms among older people, interesting patterns emerged. While digital communication demonstrated a protective effect against depressive symptoms, the influence of offline social interaction did not reach statistical significance. Additionally, integrating offline social interaction did not significantly alter the influence of digital communication on older people’s mental health. In summary, we highlight the following key conclusions. Firstly, digital communication appears to be a protective factor for older people’s mental well-being. Secondly, we did not observe a statistically significant effect of offline social interaction on older people’s mental health. Lastly, our findings suggest that the effects of digital communication and offline social interaction on depressive symptoms in older people may operate independently, without evidence for interaction between the two. The findings were also confirmed by results from PSM.

Digital communication transcends the usual boundaries of time and space, offering diverse connectivity options. This aspect of digital engagement is particularly crucial during times like the COVID-19 pandemic, as it supplements diminished physical and social interactions. It holds significant value for older people by broadening their social networks and increasing the frequency of meaningful intergenerational conversations within families. Moreover, for those facing mobility challenges or dyslexia, the versatility of digital communication platforms (through voice and video) presents effective solutions to these barriers. Vanessa Burholt’s study on older people in British communities highlights these benefits. Her research demonstrates that regular communication through texts or emails with friends and relatives can substantially mitigate the deficit in real-world social interactions. This practice significantly reduces feelings of loneliness among older adults, thereby enhancing their mental health (27).

Our examination of the impact of offline social interaction on depressive symptoms in older people revealed no statistically significant effect, a finding that diverges from previous research findings (28, 29). Despite this, we maintain a steadfast belief in the beneficial impact of offline social interaction on alleviating depressive symptoms among older adults. The disparate conclusions drawn from this study may be attributed to the unique circumstances of the study period. Specifically, the year 2020 posed unprecedented challenges for the Chinese population due to the COVID-19 pandemic. Government-imposed lockdowns and social distancing measures significantly curtailed offline social interactions. Moreover, the prohibition of mass gatherings and the stringent restrictions on public transportation capacity further limited the scope of social activities. Additionally, the pervasive focus of online media on pandemic-related news diluted the diversity of offline social engagements, potentially diminishing the protective effects of traditional social interactions on the mental health of older people.

Contrary to concerns raised by some researchers regarding the potential for excessive online engagement to displace offline interactions and exacerbate psychological issues (30), our study suggests that online and offline social interactions are not mutually exclusive and do not negatively impact depressive symptoms in older people. At least, that’s what happened during the pandemic. Drawing on Socioemotional Selectivity Theory, which posits that individuals prioritize emotionally significant relationships as they perceive their remaining time as limited (31), we propose that older adults strategically narrow their circle of social contacts. This deliberate consolidation aims to optimize the emotional returns from their constrained social resources. These intimate connections, which are often the focal point of older people’s online communications, serve as crucial emotional and support resources. The pandemic’s intermittent lockdowns and the heightened perceived risk of infection may have dampened the appeal and positive mental health effects of offline social interactions among this population. Nonetheless, the inherent advantages of online social engagement enable the continuation of close relationships, thereby safeguarding social quality for older people. Furthermore, the collective support and shared experiences during the pandemic have likely strengthened older adults’ feelings of community and emotional connection within their relationships.

### 4.1 Limitations

We acknowledge some limitations of this study, primarily related to the scope of data accessibility and the temporal relevance of our findings. Our portrayal of older people’s digital communication might not capture the full extent of their digital engagement due to limitations in the data accessible from public databases, which may exclude various aspects of their digital communication. Additionally, our analysis utilized the 2020 CHARLS database, making the results subject to temporal lag. As the digital landscape and the societal context evolve, particularly in response to the pandemic, there is a pressing need to update our understanding of the health implications of digital communication to mitigate time bias and refine the accuracy of future research outcomes. Furthermore, the applicability of our findings is entangled with the specific circumstances of the COVID-19 pandemic, which may limit the generalizability of the results to other temporal or situational contexts.

## Data Availability

'The original contributions presented in the study are included in the article/Supplementary material, further inquiries can be directed to the corresponding author.
